# Systematic Analysis of Coronavirus Disease 2019 (COVID-19) Receptor ACE2 in Malignant Tumors: Pan-Cancer Analysis

**DOI:** 10.3389/fmolb.2020.569414

**Published:** 2020-10-23

**Authors:** Jukun Song, Jing Han, Feng Liu, Xianlin Chen, Shenqi Qian, Yadong Wang, Zhenyu Jia, Xiaofeng Duan, Xiangyan Zhang, Jianguo Zhu

**Affiliations:** ^1^Department of Oral and Maxillofacial Surgery, Guizhou Provincial People’s Hospital, Guizhou, China; ^2^Department of Respiratory and Critical Care Medicine, Guizhou Provincial People’s Hospital, Guizhou, China; ^3^Department of B Ultrasound, Guizhou Provincial People’s Hospital, Guizhou, China; ^4^Department of Stomatology, Changshun County Medical Group Central Hospital, Guizhou, China; ^5^Department of Botany and Plant Sciences, University of California, Riverside, Riverside, CA, United States; ^6^Department of Urology, Guizhou Provincial People’s Hospital, Guizhou, China

**Keywords:** COVID-19, ACE2, Pan-cancer analysis, SARS-CoV-2, TCGA

## Abstract

**Background:**

Coronavirus disease 2019 (COVID-19) was first detected in patients with pneumonia in December 2019 in China and it spread rapidly to the rest of the world becoming a global pandemic. Several observational studies have reported that cancer is a risk factor for COVID-19. On the other hand, ACE2, a receptor for the SARS-CoV-2 virus, was found to be aberrantly expressed in many tumors. However, the characterization of aberrant ACE2 expression in malignant tumors has not been elucidated. Here, we conducted a systematic analysis of the ACE2 expression profile across 31 types of tumors.

**Methods:**

Distribution of ACE2 expression was analyzed using the GTEx, CCLE, TCGA pan-cancer databases. We evaluated the effect of ACE2 on clinical prognosis using the Kaplan-Meier survival plot and COX regression analysis. Correlation between ACE2 and immune infiltration levels was investigated in various cancer types. Additionally, the correlation between ACE2 and immune neoantigen, TMB, microsatellite instability, Mismatch Repair Genes (MMRs), HLA gene members, and DNA Methyltransferase (DNMT) was investigated. The frequency of ACE2 gene mutation in various tumors was analyzed. Functional enrichment analysis was conducted in various cancer types using the GSEA method.

**Results:**

In normal tissues, ACE2 was highly expressed in almost all 31 organs tested. In cancer cell lines, the expression level of ACE2 was low to medium. Although aberrant expression was observed in most cancer types, high expression of ACE2 was not linked to OS, DFS, RFS, and DFI in most tumors in TCGA pan-cancer data. We found that ACE2 expression was significantly correlated with the infiltrating levels of macrophages and dendritic cells, CD4+ T cells, CD8+ T cells, and B cells in multiple tumors. A positive correlation between ACE2 expression and immune neoantigen, TMB, and microsatellite instability was found in multiple cancers. GSEA analysis which was carried out to determine the effect of ACE2 on tumors indicated that several cancer-associated pathways and immune-related pathways were hyperactivated in the high ACE2 expression group of most tumors.

**Conclusion:**

These findings suggest that ACE2 is not correlated with prognosis in most cancer types. However, elevated ACE2 is significantly correlated with immune infiltrating levels, including those of CD8+ T cells, CD4+ T cells, macrophages, neutrophils, and DCs in multiple cancers, especially in lung and breast cancer patients. These findings suggest that ACE2 may affect the tumor environment in cancer patients with COVID-19.

## Introduction

Coronavirus disease 2019 (COVID-19) is an acute pneumonia caused by Severe Acute Respiratory Syndrome Coronavirus 2 (SARS-CoV-2) infection. Since the public report of a SARS-CoV-2 infection in China in December 2019, COVID-19 has spread rapidly to many countries causing a global pandemic (Chen et al., 2020; Xu X. W. et al., 2020). The World Health Organization (WHO), therefore, announced a global health emergency on January 30, 2020 (Eurosurveillance editorial team, 2020). As of April 2, 2020, the new virus infection has caused 82,735 and 855,446 cumulative COVID-19 cases in China and globally, with 3,327 and 43,955 deaths reported, respectively.

Accumulating evidence demonstrates that the Spike (S) protein of SARS-CoV-2 binds Angiotensin-Converting Enzyme 2 (ACE2) to trigger COVID-19 (Hoffmann et al., 2020). Besides, the expression of ACE2 is a key determinant for the entry of SARS-CoV-2 into host cells (Hamming et al., 2004; Kuba et al., 2005). Several studies report that the expression and distribution of ACE2 are enriched in the lungs (Lukassen et al., 2020), esophagus (Qi et al., 2020), kidneys (Pan et al., 2020), bladder (Zou et al., 2020), testis (Wang and Xu, 2020), stomach (Qi et al., 2020), ileum (Zou et al., 2020), and oral mucosa (Xu H. et al., 2020) using a single-cell RNA sequencing technique. These findings suggested that organs with high ACE2 expression are potentially high risk for SARS-CoV-2 infection. Cancer patients in a state of systemic immunosuppression, are considered to be highly vulnerable to the COVID-19 epidemic (Yang et al., 2020). Current clinical studies show that COVID-19 patients with tumors have a higher risk of clinical complications and death than patients without cancer (Liang et al., 2020; Yu et al., 2020; Zhang et al., 2020). However, a systematic analysis of the aberrant expression of ACE2 in human cancer has not yet been conducted. Therefore, in this study a bioinformatics approach was used to evaluate the prognosis of the ACE2 in TCGA Pan-cancer data. Furthermore, the association between ACE2 expression and immune neoantigen, TMB, microsatellite instability, and HLA family members was investigated.

## Materials and Methods

### Transcriptome Data

Gene expression profiles were downloaded from the three publicly available datasets, the TCGA Pan-cancer cohort^1^, Genotype-Tissue Expression (GTEx) project^2^, and Broad Institute Cancer Cell Line Encyclopedia (CCLE)^3^. mRNA data in normal tissues were obtained from the GTEx project, which included 31 tissues. Distribution of the expression levels of cancer cell lines in 21 organizations was also conducted. The mRNA data of adjacent tumor tissues and tissue in 31 types of tumors were obtained from the TCGA dataset. Clinical information was also obtained (Liu et al., 2018) and the Kruskal-Wallis test was done to determine the differences among organs.

### Differentially Expressed Analysis

To determine the difference between normal healthy tissues, adjacent tumor samples, and tumor samples, TCGA Pan-cancer and GTEx datasets were downloaded from the UCSC XENA dataset^4^. The tumor samples and adjacent samples were obtained from the TCGA dataset while the normal samples were obtained from the GTEx dataset. The difference in ACE expression was compared between normal healthy tissues and tumor tissues and also between adjacent tumor tissues and tumor tissues. The Wilcox.test was used to calculate the significance of difference with a threshold of *P* < 0.05.

### Prognostic Analysis in Pan-Cancer Levels

To evaluate whether the ACE2 expression level was associated with tumor prognosis in various cancers, univariate COX regression analysis was performed for Overall Survival (OS), Disease-Free Survival (DFS), Disease-Specific Survival (DSS), Disease-Free Interval (DFI), and Progression-Free Interval (PFI). The threshold was adjusted to a Cox *P* < 0.05. Based on the median of ACE2 expression levels, samples were divided into two groups: the high expression and the low expression group. Kaplan-Meier survival analysis was used to compare the differences in 31 types of tumors. A log-rank test was used to calculate the significance of survival differences with a threshold of *P* < 0.05. We also evaluated the association between ACE2 expression level and other clinical features, including the TNM stage.

### Relationship Between ACE2 Expression Level and Immunity in 31 Types of Tumors

Tumor-infiltrating lymphocytes are independent predictors of cancer sentinel lymph node status and survival (Jin and Hu, 2020; Koletsa et al., 2020). We investigated whether the expression of ACE2 is related to the levels of immune infiltration in different types of cancers. The CIBERSORT method was employed to evaluate the relative proportion of 22 immune cell infiltrations across multiple cancers (Newman et al., 2015). Meanwhile, the ESTIMATE method was used to assess the immune cell infiltration levels including the immune score, tumor purity, and stromal score for each tumor sample in the TCGA pan-cohort (Yoshihara et al., 2013). The correlation between ACE2 and scores of these immune cells in 31 types of cancers was analyzed using the Spearman correlation method.

Under normal circumstances, the immune system can recognize and remove tumor cells in the tumor microenvironment. Tumor immunotherapy is a treatment method to control and eliminate tumor cells by restarting and maintaining the tumor’s immune cycle thus restoring the normal anti-tumor immune response in the body. The immune checkpoint genes include monoclonal antibody class immune checkpoint inhibitors, therapeutic antibodies, cancer vaccines, cell therapy, and small molecule inhibitors (Danilova et al., 2019). We collected more than forty immune checkpoint genes and analyzed the relationship between ACE2 gene expression and the expression of immune checkpoint genes using the Spearman correlation analysis.

### Association Between ACE2 and Immune Neoantigen, TMB, Microsatellite Instability (MSI), HLA Family Members

Neoantigen is a neonatal antigen that is encoded by a mutant gene of a tumor cell. Different from the proteins expressed by normal cells, it is a new abnormal protein, mainly resulting from a point mutation, deletion mutation, or gene fusion (Srivastava et al., 2020). The peptide fragments formed from these proteins after enzymatic hydrolysis are presented as antigens by DC cells to T cells. DC cells promote the activation of T cells to mature T cells that specifically recognize new tumor antigens and also promote the proliferation of the activated T cells (Rooney et al., 2015). Neoantigen vaccines can be developed using the immune activity of tumor neoantigens, according to a specific mutation in the tumor cells, then administered to patients to achieve the required therapeutic effect. Therefore, the number of neonatal antigens in each tumor sample was imputed and analyzed. The correlation between ACE2 expression and the gene markers of immune neoantigen was analyzed using the Spearman correlation method.

The Tumor Mutational Burden (TMB) is usually measured by the number of somatic mutations that occur within an average of 1 Mb in the coding region (exon region) of the tumor cell genome (non-synonymous mutations) (Krieger et al., 2020). The total number of synonymous mutations indicate that the mutation pattern includes single nucleotide mutation (SNV) and small fragment insertion/deletion (Indel) and other forms of mutation. TMB is used to reflect the number of mutations in tumor cells and is a quantifiable biomarker. Here, we counted the TMB of each tumor sample separately and analyzed the relationship between gene expression and TMB using the Spearman rank correlation coefficient.

Microsatellite Instability (MSI) refers to any change in the length of a microsatellite due to the insertion or deletion of a repeating unit in a tumor compared to normal tissue. The appearance of a new microsatellite allele is a genetic phenomenon (Bonneville et al., 2017). We analyzed the correlation between gene expression and MSI using the Spearman rank correlation coefficient.

Disparities in the expression of HLA (Human Leukocyte Antigen) family members on the surface of tumor cells are part of the early and common activities that promote carcinogenesis. This is because HLA is essential for the immune recognition of tumor cells and signal transmission between tumor and immune cells (Campoli and Ferrone, 2008; Sim and Sun, 2017). Thus, we also investigated the correlation between ACE2 expression and HLA genes.

### Mutation Pattern of ACE2 Gene in Various Tumor Samples

We downloaded mutation data of multiple malignancies from the TCGA database and analyzed the alterations of the ACE2 gene within these tumors. We visualized the tumor with the most ACE2 mutations using an R data package, maftools (Mayakonda and Koeffler, 2016).

### Correlation Between ACE2 Expression Level and Mismatch Repair Genes (MMRs) and DNA Methyltransferase (DNMT)

Mismatch Repair Genes (MMRs) are the key players in the intracellular mismatch repair mechanism (Meiser et al., 2020). Loss of a key gene function in this mechanism can lead to DNA replication errors that cannot be repaired and in turn, lead to more somatic mutations. Therefore, TCGA expression profiling was used to evaluate the relationship between ACE2 and five MMRs genes: MLH1, MSH2, MSH6, PMS2, and EPCAM mutations.

DNA methylation is a chemical modification of DNA, which changes the genetic activity without changing the DNA sequence. DNA methylation causes changes in the chromatin structure, DNA conformation, DNA stability, and the proteins-DNA interaction, thereby regulating gene expression. In DNA methylation, a methyl group is covalently bound to the 5 carbon of cytosine, of the genomic CpG dinucleotide, under the catalysis of DNA methyltransferase (Yu et al., 2019). Here, we analyzed the correlation between gene expression and the expression of four methyltransferases (DNMT1, DNMT2, DNMT3A, and DNMT3B).

### GSEA Analysis Across TCGA Pan-Cancer

To determine the effect of ACE2 expression on tumors, we divided the samples into high and low expression groups according to their gene expression level and used Gene Set Enrichment Analysis (GSEA) (Barber et al., 2006) to analyze the enrichment of KEGG and HALLMARK pathways in the high and low expression groups. The gene sets using the c5 curated signatures were downloaded from the Molecular Signature Database (MSigDB) of the Broad Institute. The KEGG terms were defined between the high ACE2 expression group and the low ACE2 expression group. The significant enrichment of pathways was determined based on FDR < 0.05.

## Results

### High ACE2 Expression in the Organs of a Healthy Population

**FIGURE 1:**
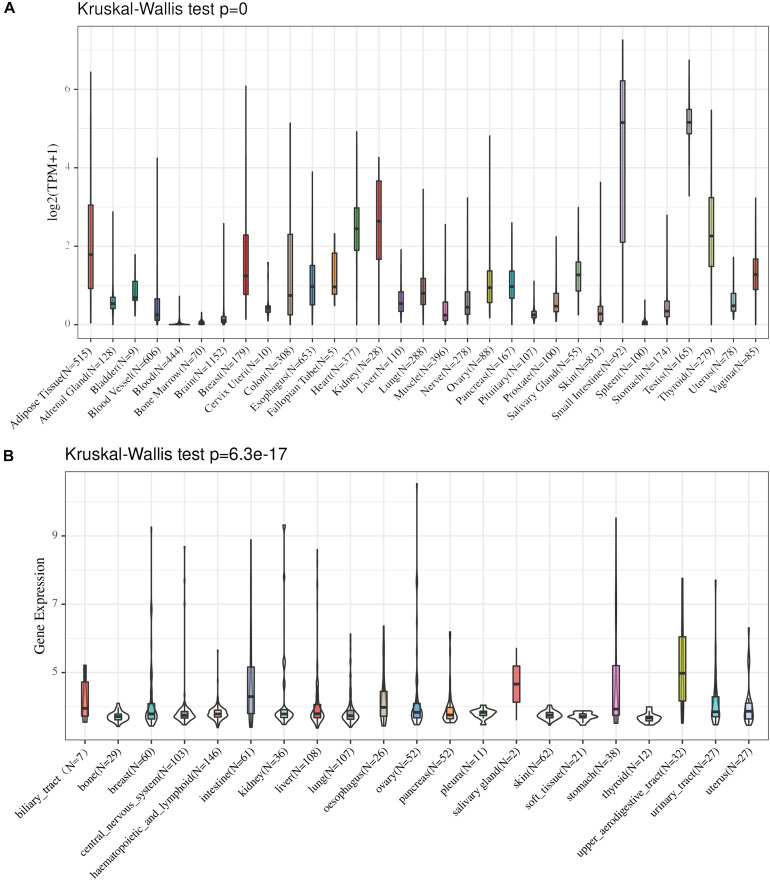
The distribution of ACE expression in normal organs in the GTEx dataset **(A)** and CCLE dataset **(B)**.

We analyzed the distribution of ACE2 in the GTEx dataset, and the results indicated that ACE2 is highly expressed in most of the organs tested. The highest expression levels were noted in the small intestine, testis, and thyroid while the lowest expression levels were in the blood, brain, and breast (Figure 1A). The Kruskal-Wallis test indicated that there were significant differences in the ACE2 expression levels among the organs.

### Low to Medium Expression of ACE2 in Cancer Cell Lines

The distribution of ACE2 in cancer cell lines was analyzed, and our findings showed that the expression level of ACE2 in cancer cell lines was low to medium in different tissues. The highest expression levels were noted in the intestine, salivary gland, and upper aerodigestive tract while the lowest expression levels were in the bone, pleura, and soft tissue (Figure 1B). The Kruskal-Wallis test indicated that there were significant differences in the ACE2 expression levels among the organs.

### ACE2 mRNA Expression in Different Types of Human Cancers

**FIGURE 2:**
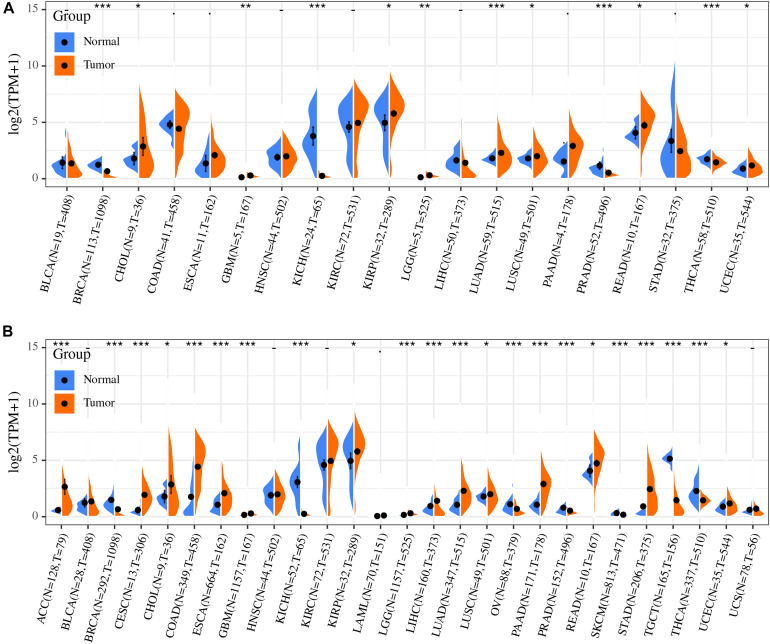
Differential expression analysis revealed that ACE2 expression was significantly different between primary tumor and solid tissue normal samples **(A)**, normal samples, and tumor samples **(B)**. *P < 0.05, **P < 0.01; ***P < 0.001.

The distribution of ACE2 expression in the TCGA pan-cancer dataset is shown in Figure 2A. Differences in the ACE2 expression level between the primary tumor and adjacent tumor samples were observed in multiple tumor types. ACE2 expression was upregulated in seven tumors types (CHOL, GBM, KIRP, LGG, LUAD, LUSC, READ, and UCEC) and downregulated in six tumors types (BRCA, KICH, LIHC, PRAD, STAD, and THCA). However, no significant difference was observed in ACE2 expression in BLCA, COAD, ESCA, HNSC, KIRC, LIHC, PAAD, and STAD.

Considering that there are relatively few tumor samples in the TCGA database, we combined the data of normal tissues from the GTEx database with the data of TCGA tumor tissues to analyze the ACE2 expression differences in 31 types of tumors. Our results indicate that there were significant differences in ACE2 expression in multiple tumors (Figure 2B). This analysis showed that ACE2 expression was higher in colorectal, gastric, kidney, lung, esophagus, pancreatic cancers, and lymphoma tumors compared to the normal tissues. Lower ACE2 expression was also observed in the breast, testis, thyroid, skin, ovary, and prostate tumors in some data sets.

### Prognostic Analysis in TCGA Pan-Cancer Level

**FIGURE 3:**
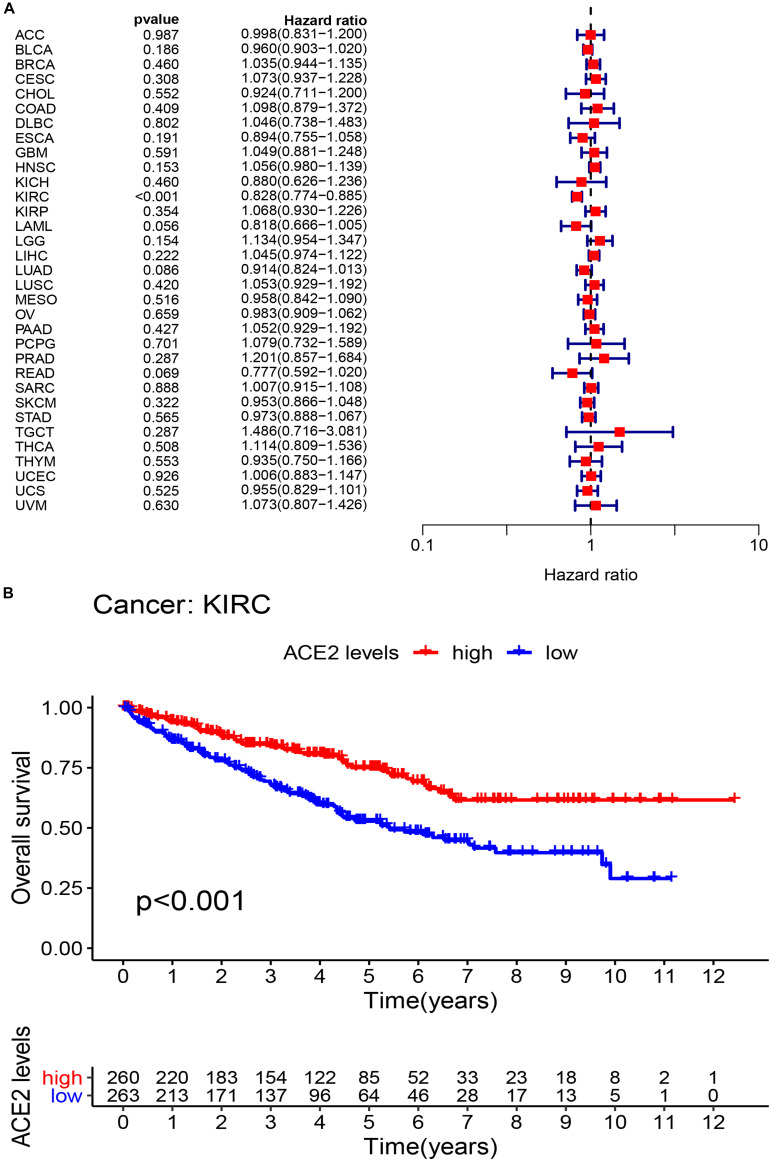
The overall survival (OS) rates across 31 types of tumors. **(A)** The univariate Cox regression analysis exhibiting OS. **(B)** Kaplan-Meier survival curves showing the association of high and low expression of ACE2 with survival rates in KIRC.

We investigated whether ACE2 expression was linked with Overall Survival (OS) in cancer patients. Using univariate COX regression analysis, the forest plot in multiple cancer types is shown in Figure 3A and Table 1. The results demonstrated that ACE2 has less prognostic influence on most cancers, except for KIRC (OS HR = 0.83, CI = 0.77–0.89, *P* < 0.001). Kaplan-Meier survival analysis was performed to screen for prognostic tumor types. The prognostic Kaplan-Meier survival curve is shown in Figure 3B. Higher ACE2 expression was associated with improved OS in KIRC. The results were linked with the COX results, which also indicated that ACE2 expression was not related to prognosis in most tumors.

**TABLE 1:** Results of univariate Cox regression analysis showing the overall survival of 31 types of tumors.

Type of tumor	HR	HR.95L	HR.95H	*P*-value
ACC	0.998466	0.830981	1.199707	0.986922
BLCA	0.959891	0.903359	1.01996	0.186234
BRCA	1.035286	0.94435	1.134979	0.459732
CESC	1.072815	0.937188	1.22807	0.308086
CHOL	0.923638	0.711122	1.199665	0.551562
COAD	1.098232	0.879351	1.371595	0.408667
DLBC	1.04575	0.737658	1.482521	0.801644
ESCA	0.893723	0.755066	1.057843	0.191473
GBM	1.048813	0.881289	1.248182	0.59144
HNSC	1.056246	0.979796	1.138662	0.153433
KICH	0.879568	0.625816	1.23621	0.459953
KIRC	0.827512	0.773639	0.885137	3.54*E*−08
KIRP	1.067562	0.929821	1.225708	0.353621
LAML	0.818356	0.666267	1.005163	0.056021
LGG	1.133532	0.954175	1.346604	0.15381
LIHC	1.045119	0.973617	1.121871	0.222275
LUAD	0.913706	0.824287	1.012824	0.085895
LUSC	1.052555	0.929319	1.192133	0.42013
MESO	0.958148	0.842263	1.089978	0.515679
OV	0.982709	0.909402	1.061925	0.659244
PAAD	1.051899	0.928528	1.191661	0.426658
PCPG	1.078945	0.732458	1.589338	0.700614
PRAD	1.201496	0.85705	1.684373	0.286876
READ	0.776572	0.591509	1.019536	0.068659
SARC	1.006876	0.914899	1.1081	0.888498
SKCM	0.952981	0.866334	1.048293	0.322058
STAD	0.9734	0.887942	1.067083	0.565259
TGCT	1.485636	0.716476	3.080516	0.287383
THCA	1.114409	0.808556	1.535958	0.508125
THYM	0.935351	0.75005	1.166429	0.552972
UCEC	1.006279	0.882521	1.147393	0.925516
UCS	0.954994	0.828676	1.100566	0.524662
UVM	1.072607	0.8066	1.426341	0.629808

**FIGURE 4:**
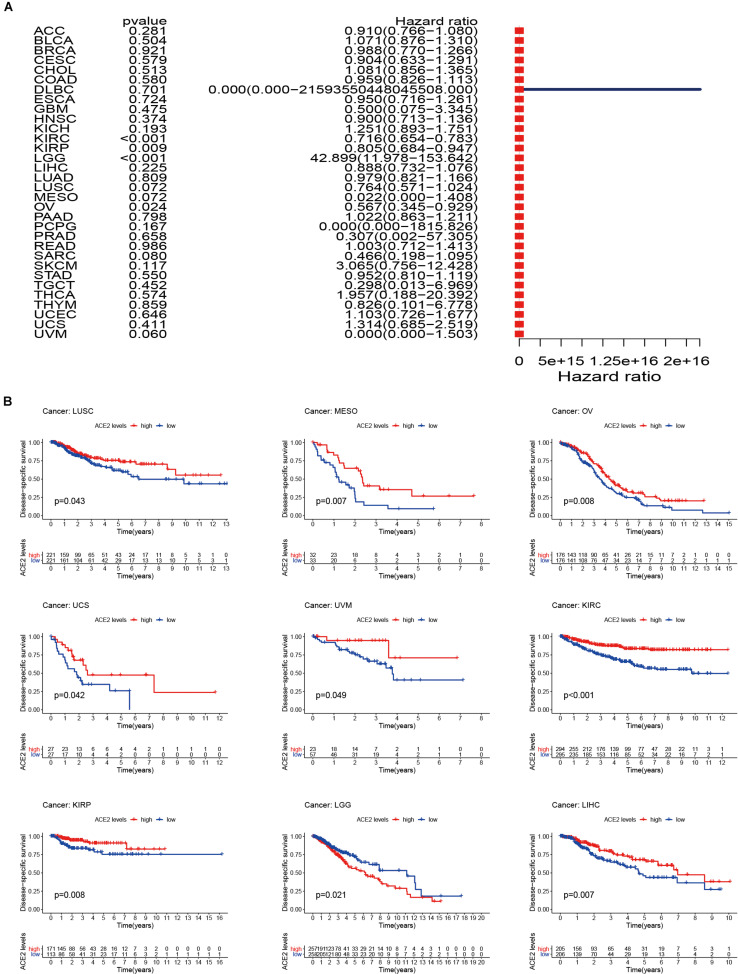
Disease-specific survival (DSS) of 31 types of tumors. **(A)** The results of univariate Cox regression analysis showing the DSS. **(B)** Kaplan-Meier survival curves comparing the survival rates of high and low expression of ACE2 in several tumors.

Considering that there may be non-tumor factors leading to death during the follow-up period, we further analyzed the relationship between ACE2 expression and prognosis Disease-Specific Survival (DSS) among 31 types of tumors. The results indicated that ACE2 was not correlated with DSS in most tumors, except for KIRC (DDS HR = 0.65, CI = 0.65–0.78, *P* < 0.001), LGG (DDS HR = 11.97, CI = 11.97–153.64, *P* < 0.001), KIRP (DDS HR = 0.81, CI = 0.68–0.0.95, *P* < 0.001) and OV (DDS HR = 0.57, CI = 0.35–0.93, *P* < 0.001) (Figure 4A and Table 2). Kaplan-Meier survival analysis showed that there were significant differences in ACE2 expression in eight types of cancers (KIRC, KIRP, LGG, LIHC, LUSC, OV, UCS, and UVM) (Figure 4B). Among these cancers, higher ACE2 was associated with better DSS, except for LGG.

**TABLE 2:** Results of univariate Cox regression analysis showing the disease-specific survival of 31 types of tumors.

Type of tumor	HR	HR.95L	HR.95H	*P*-value
ACC	0.909923	0.766369	1.080368	0.281236
BLCA	1.07117	0.875662	1.310327	0.503721
BRCA	0.987528	0.770341	1.265947	0.921107
CESC	0.903873	0.632753	1.291161	0.578571
CHOL	1.081106	0.855946	1.365496	0.51279
COAD	0.958759	0.82588	1.113018	0.580073
DLBC	0.000104	5.02*E*−25	2.16*E*+16	0.700829
ESCA	0.950325	0.716226	1.26094	0.724007
GBM	0.499958	0.07473	3.344822	0.474691
HNSC	0.899736	0.712743	1.135787	0.374098
KICH	1.250501	0.893255	1.750623	0.192804
KIRC	0.715883	0.654247	0.783325	3.43*E*−13
KIRP	0.805045	0.684349	0.947028	0.008878
LGG	42.89862	11.9778	153.6419	7.71*E*−09
LIHC	0.887651	0.732186	1.076125	0.225077
LUAD	0.978579	0.821253	1.166042	0.808664
LUSC	0.764446	0.570636	1.024082	0.071788
MESO	0.022229	0.000351	1.407965	0.072127
OV	0.566552	0.345418	0.929255	0.024411
PAAD	1.022364	0.863185	1.210897	0.797844
PCPG	1.54*E*−08	1.31*E*−19	1815.826	0.166669
PRAD	0.307207	0.001647	57.30509	0.65819
READ	1.003112	0.71232	1.412615	0.985807
SARC	0.465645	0.198031	1.094906	0.079753
SKCM	3.064603	0.755684	12.42821	0.116928
STAD	0.951974	0.81024	1.118501	0.549579
TGCT	0.298159	0.012757	6.968862	0.451704
THCA	1.957482	0.187905	20.39188	0.574293
THYM	0.825962	0.100657	6.777603	0.858686
UCEC	1.103095	0.725522	1.677162	0.646238
UCS	1.313979	0.685276	2.519483	0.411014
UVM	8.97*E*−05	5.35*E*−09	1.502875	0.060398

**FIGURE 5:**
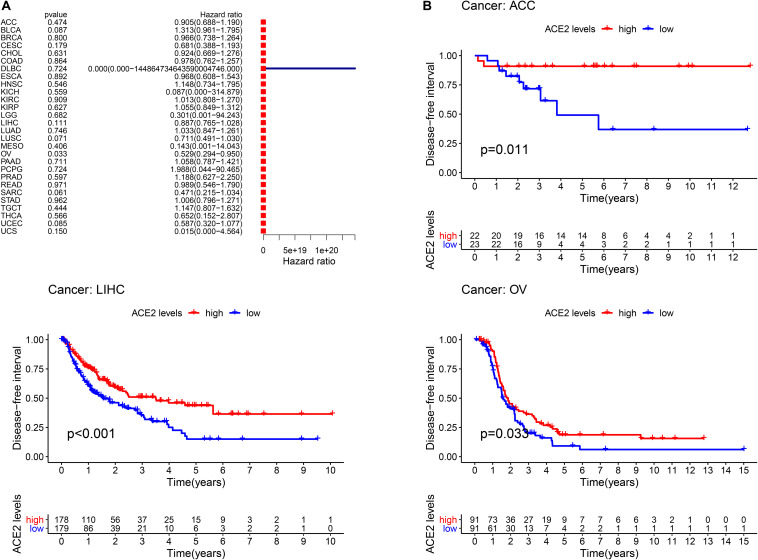
Comparison of the disease-free interval (DFI) analysis across the 31 types of tumors. **(A)** Results of univariate Cox regression analysis for DFI. **(B)** Kaplan-Meier survival curves comparing the association of high and low expression of ACE2 with the survival rates in several tumors.

Furthermore, we analyzed the correlation between ACE2 expression and prognosis Disease-Free Interval (DFI) in 31 types of tumors. The forest plot is shown in Figure 5A and Table 3. In most tumors except for OV (DFI HR = 0.53, CI = 0.29–0.95, *P* < 0.001), ACE2 was not associated with DFI. Kaplan-Meier survival analysis showed that there were significant differences in ACE2 expression in ACC, LIHC, and OV (Figure 5B).

**TABLE 3:** Results of univariate Cox regression analysis showing the disease-free interval of 31 types of tumors.

Type of tumor	HR	HR.95L	HR.95H	*P*-value
ACC	0.904678	0.687715	1.190089	0.473967
BLCA	1.313151	0.960902	1.794528	0.087326
BRCA	0.965821	0.738143	1.263725	0.799853
CESC	0.680629	0.388157	1.193476	0.179369
CHOL	0.923904	0.668967	1.275996	0.630907
COAD	0.978421	0.761775	1.256682	0.86436
DLBC	3.77*E*−05	9.79*E*−30	1.45*E*+20	0.724317
ESCA	0.968343	0.607753	1.542876	0.892331
HNSC	1.147606	0.733682	1.795056	0.546378
KICH	0.08696	2.40*E*−05	314.8793	0.559117
KIRC	1.013184	0.808404	1.269837	0.909479
KIRP	1.055433	0.849028	1.312017	0.627027
LGG	0.300646	0.000959	94.24319	0.681937
LIHC	0.886907	0.76511	1.028093	0.111301
LUAD	1.033448	0.847177	1.260675	0.745593
LUSC	0.711268	0.491238	1.029853	0.071201
MESO	0.142899	0.001454	14.04333	0.405862
OV	0.528736	0.294211	0.95021	0.033112
PAAD	1.057515	0.786871	1.421246	0.710809
PCPG	1.988029	0.043688	90.46544	0.724268
PRAD	1.188051	0.627316	2.250008	0.596914
READ	0.98896	0.546375	1.790055	0.970747
SARC	0.471045	0.214532	1.034267	0.060655
STAD	1.005747	0.795938	1.270862	0.961709
TGCT	1.147477	0.806776	1.632054	0.444047
THCA	0.652405	0.151606	2.807489	0.566248
UCEC	0.586972	0.319981	1.076739	0.08523
UCS	0.015272	5.11*E*−05	4.563611	0.15045

**FIGURE 6:**
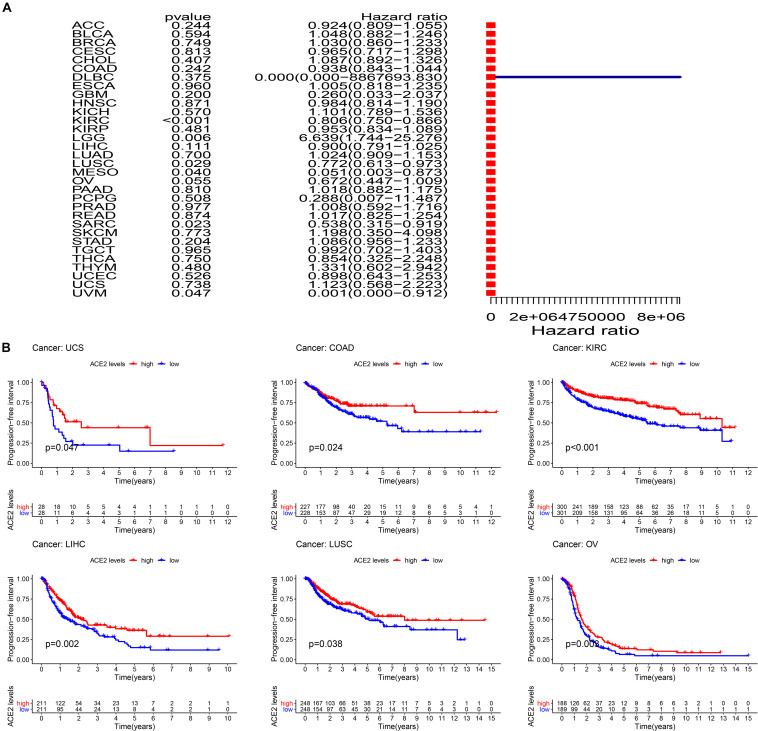
Comparison of the progression-free interval (PFI) across 31 types of tumors. **(A)** Results of univariate Cox regression analysis showing the PFI. **(B)** Kaplan-Meier survival curves showing the association of the high and low expression of ACE2 with the survival rates of several tumors.

Additionally, we analyzed the relationship between ACE2 expression and Progression-Free Interval (PFI) in multiple cancer types. The summarized forest plot is shown in Figure 6A and Table 4. In most tumors ACE2 was not associated with PFI except for KIRC (PFI HR = 0.81, CI = 0.75–0.87, *P* < 0.001), LGG (PFI HR = 6.64, CI = 1.74–25.28, *P* < 0.001), SARC (PFI HR = 0.54, CI = 0.31–0.92, *P* < 0.001), LUSC (PFI HR = 0.77, CI = 0.61–0.97, *P* < 0.001), and MESO (PFI HR = 0.05, CI = 0.003–0.873, *P* < 0.001). Kaplan-Meier survival analysis showed that there were significant differences in ACE2 expression in COAD, KIRC, LIHI, LUSC, OV, and USC (Figure 6B).

**TABLE 4:** Results of univariate Cox regression analysis showing the progression-free interval of 31 types of tumors.

Type of tumor	HR	HR.95L	HR.95H	*P*-value
ACC	0.924098	0.809098	1.055442	0.244357
BLCA	1.048174	0.881605	1.246213	0.594138
BRCA	1.029772	0.86016	1.232829	0.749351
CESC	0.964894	0.717139	1.298243	0.813405
CHOL	1.087358	0.891976	1.325537	0.407244
COAD	0.938018	0.842681	1.044141	0.241968
DLBC	1.77*E*−06	3.52*E*−19	8867694	0.374656
ESCA	1.005252	0.818361	1.234822	0.960194
GBM	0.260066	0.033209	2.036611	0.199634
HNSC	0.984371	0.814243	1.190044	0.870739
KICH	1.101151	0.78947	1.535883	0.570337
KIRC	0.805519	0.749562	0.865654	3.92*E*−09
KIRP	0.953315	0.83449	1.089059	0.481492
LGG	6.639018	1.743826	25.27578	0.005516
LIHC	0.900164	0.790906	1.024516	0.111137
LUAD	1.02375	0.908678	1.153395	0.699622
LUSC	0.772233	0.612712	0.973286	0.028573
MESO	0.051477	0.003034	0.873402	0.040008
OV	0.671693	0.447005	1.009321	0.055453
PAAD	1.017794	0.88173	1.174854	0.809641
PCPG	0.287896	0.007215	11.48711	0.507958
PRAD	1.007748	0.591765	1.716147	0.977331
READ	1.01708	0.824779	1.254217	0.874154
SARC	0.537535	0.314556	0.918577	0.02317
SKCM	1.198481	0.350463	4.098451	0.772881
STAD	1.085933	0.956114	1.233379	0.204404
TGCT	0.992281	0.701739	1.403116	0.965032
THCA	0.854497	0.324754	2.248363	0.75006
THYM	1.330682	0.601975	2.941509	0.480247
UCEC	0.897706	0.643179	1.252957	0.52585
UCS	1.123456	0.567845	2.222707	0.738087
UVM	0.001209	1.60*E*−06	0.911775	0.046891

The association of ACE2 with the TNM stage across 31 types of tumors was performed. Results showed no correlation between ACE2 and the TNM stage in all tumors (Supplementary Figure 1).

### ACE2 Expression Is Correlated With Immune Infiltration Levels in TCGA Pan-Cancers

**FIGURE 7:**
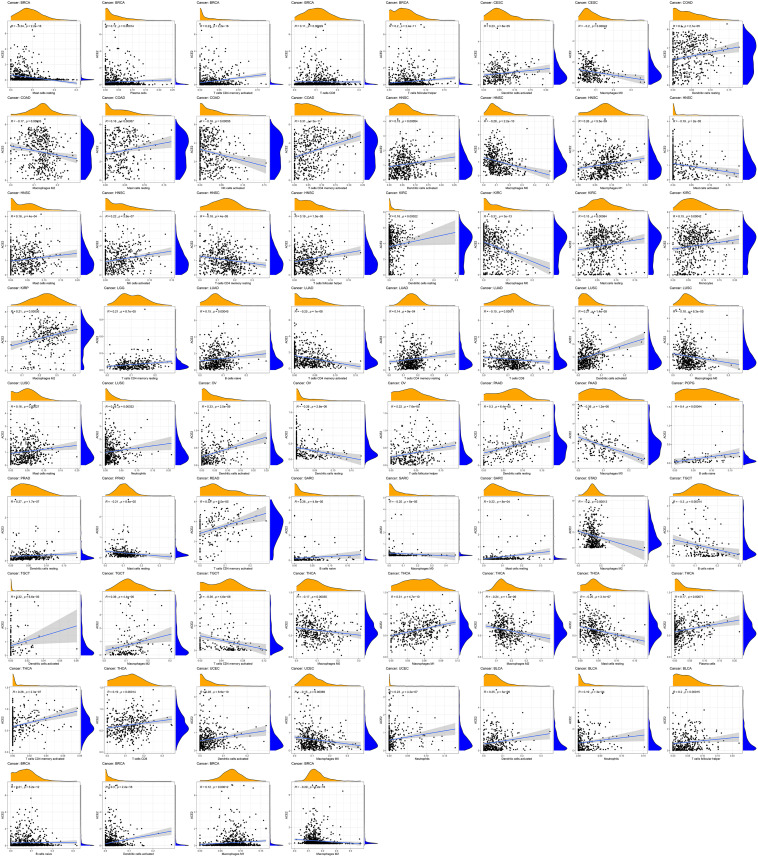
Correlation of ACE2 expression with immune infiltration level in COAD (colon adenocarcinoma), LUSC (lung squamous cell carcinoma), and STAD (stomach adenocarcinoma).

We employed the CIBERSORT algorithm to determine the relative fractions of immune cell infiltration in various tumors. We found that ACE2 expression was correlated with immune infiltration levels in different types of cancers. ACE2 expression was significantly correlated with: macrophages infiltration levels in 13 types of cancers; dendritic cells in 12 types of cancers; CD4+ T cells in eight types of cancers; CD8+ T cells in three types of cancers; mast cells in eight types of cancers; B cells in five types of cancers; and NK cells in two types of cancers (Figure 7). For example, ACE2 expression level in LUSC was linked with infiltration levels of dendritic cells (*r* = 0.27, *P* = 1.4e-09), macrophages M0 (*r* = -0.18, *P* = 6.3e-05), mast cells resting (*r* = 0.16, *P* = 0.00027), and neutrophils (*r* = 0.16, *P* = 0.00032).

**FIGURE 8:**
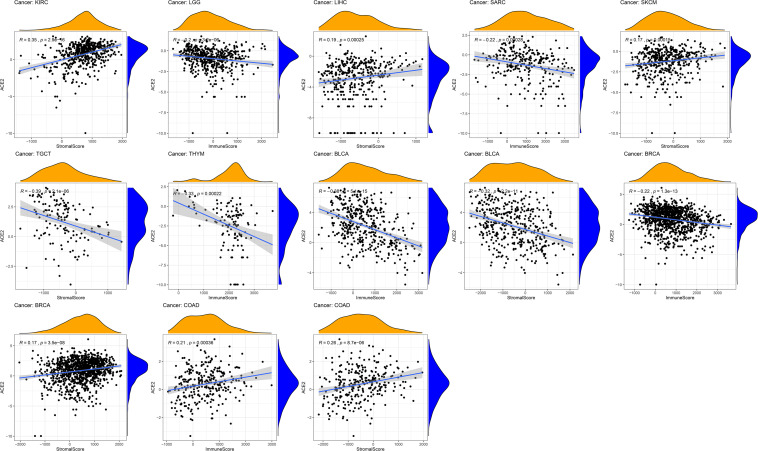
Correlation of ACE2 expression with the immune score, tumor purity, and stomal score in COAD (colon adenocarcinoma), LUSC (lung squamous cell carcinoma), and STAD (stomach adenocarcinoma).

Given the correlation of ACE2 expression with immune infiltration levels in different types of cancers, we analyzed the association between ACE2 and tumor purity. We found that ACE2 expression positively correlates with immune infiltration in BLCA and BRCA, LGG, SARC, TGCT, THYM, while a negative association was observed in COAD and KIRC, LIHC, SKCM (Figure 8). These findings strongly suggest that ACE2 plays a vital role in immune infiltration in several tumors.

**FIGURE 9:**
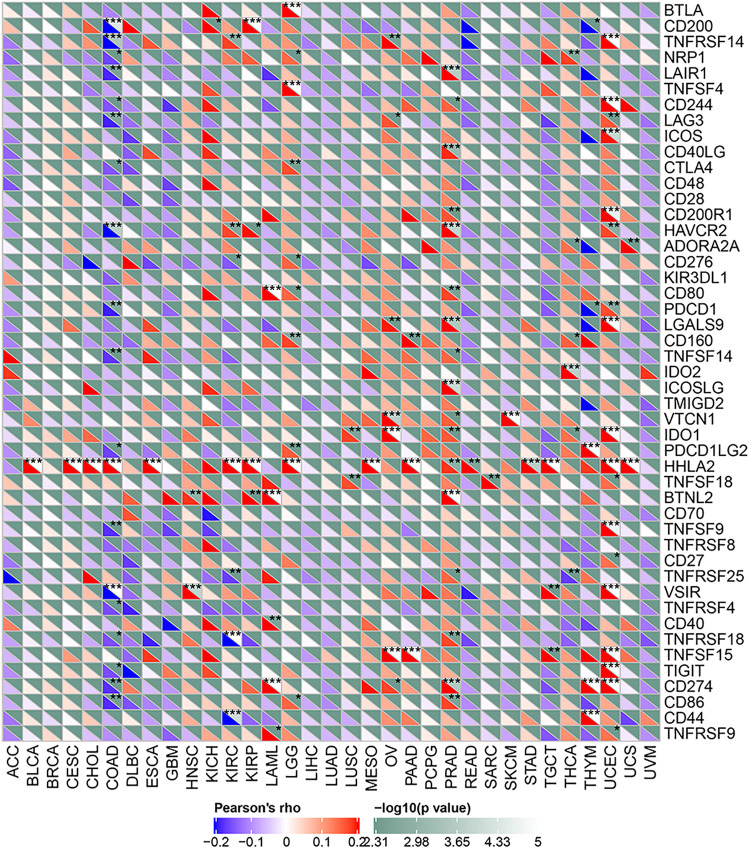
A heatmap showing the relationship between ACE2 and the expression of immune checkpoint genes. *P < 0.05, **P < 0.01; ***P < 0.001.

Tumor immunotherapy is a recent approach to treating malignant tumors. We collected more than forty immune checkpoint genes and analyzed their relationship with ACE2 expression. A correlation heatmap showing the association of the immune checkpoint genes and ACE2 expression is shown in Figure 9. We found that ACE2 expression correlates with the immune checkpoint gene in PRAD, COAD, and UCEC.

### Immune Neoantigen, TMB, HLA, and Microsatellite Instability

**FIGURE 10:**
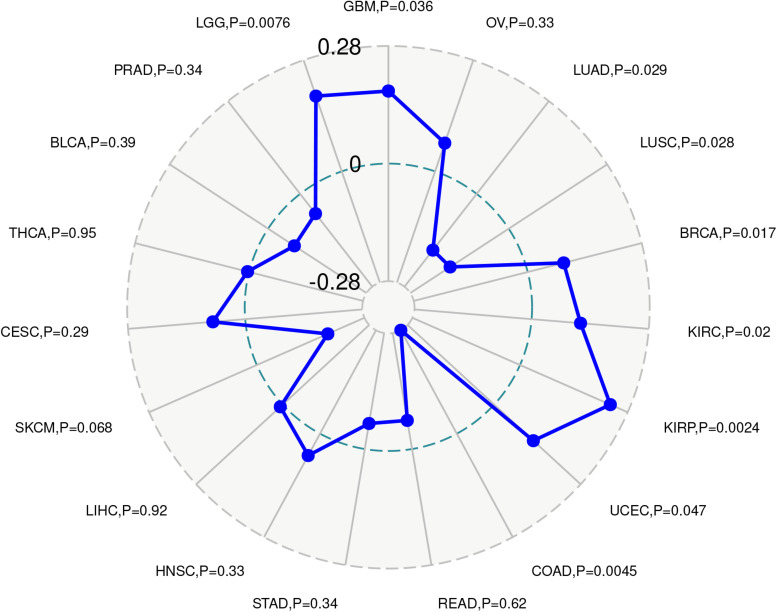
Relationship between ACE2 expression level and the tumor neoantigens.

We regularly collect and synthesize neoantigen vaccines, according to mutations in specific tumor cells, and using the immune activity of tumor neoantigens we immunize patients to achieve the required therapeutic effect. Therefore, we counted the number of new antigens in each tumor sample and analyzed the relationship between ACE2 expression and the number of antigens. The ACE2 expression level was positively associated with immune neoantigen in GBM, LGG, BRCA, KIRC, and KIRP while in the LUAD, COAD, LUSC, and UCEC, a negative relationship was observed (Figure 10).

**FIGURE 11:**
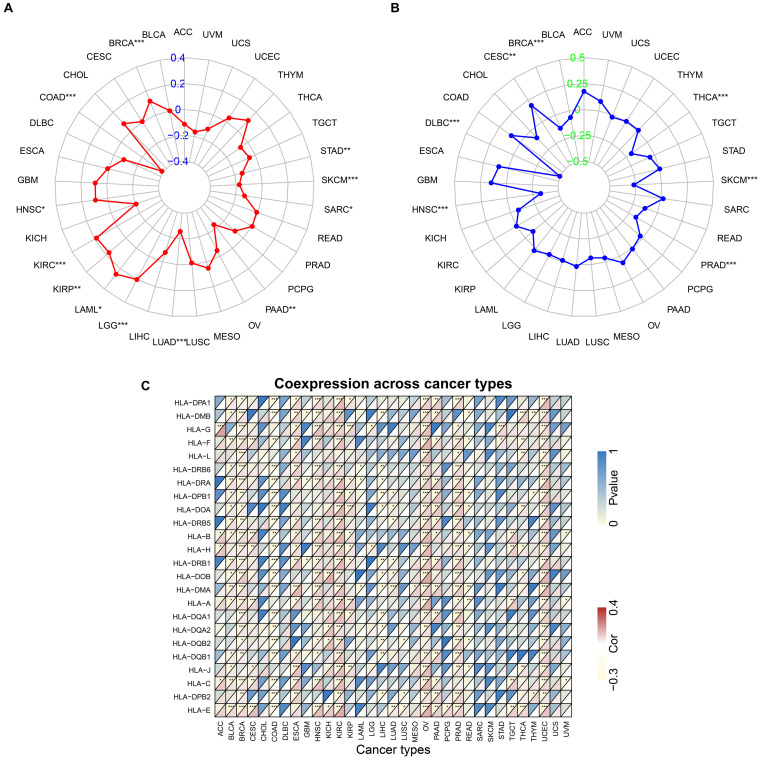
Correlation between ACE2 expression level and TMB **(A)** and Microsatellite instability **(B)**, HLA genes **(C)**. *P < 0.05, **P < 0.01; ***P < 0.001.

TMB reflects the number of mutations in tumor cells and is a quantifiable biomarker. Therefore, the TMB of each tumor sample was counted separately and the relationship between ACE2 expression and TMB was analyzed using the Spearman rank correlation coefficient. Significant correlations were found between ACE2 expression and TMB in BRCA, STAD, SKCM, COAD, SARC, HNSC, KIRC, KIRP, LAML, LGG, LUAD, and PAAD (Figure 11A). These results indicate that there is a strong relationship between ACE2 and TMB in most tumors.

Microsatellite instability (MSI) is a pattern of hypermutation that occurs in genomic microsatellites and is caused by defects in the mismatch repair system. The correlation between ACE2 and MSI was analyzed using the Spearman rank correlation coefficient. Results showed a significant correlation between ACE2 expression and MSI in BRCA, CESC, DLBC, THCA, SKCM, HNSC, and PRAD (Figure 11B). These results revealed a strong relationship between ACE2 and MSI in most of the tumors. Notably, many HLA genes showed a significant correlation with ACE2 expression in most tumors (Figure 11C).

### Identification of Gene Mutation Patterns in Pan-Cancer

**FIGURE 12:**
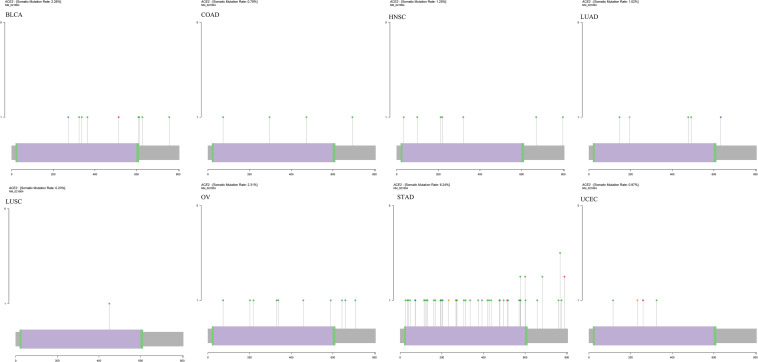
Identification of gene mutation patterns in several cancers.

The mutation data of 31 tumors were downloaded from TCGA, and we analyzed the ACE2 mutation in these tumors. A schematic diagram of the tumor with the highest number of mutations is shown in Figure 12. Notably, ACE2 mutations were only observed in BLCA, BRCA, COAD, HNSC, LAML, LGG, LUAD, LUSC, OV, SKCM, STAD, and UCEC. The highest mutation was in UCEC. These findings indicate that ACE2 mutations seldom occur in most tumors.

### Mismatch Repair Genes (MMRs) and DNA Methyltransferase (DNMT)

**FIGURE 13:**
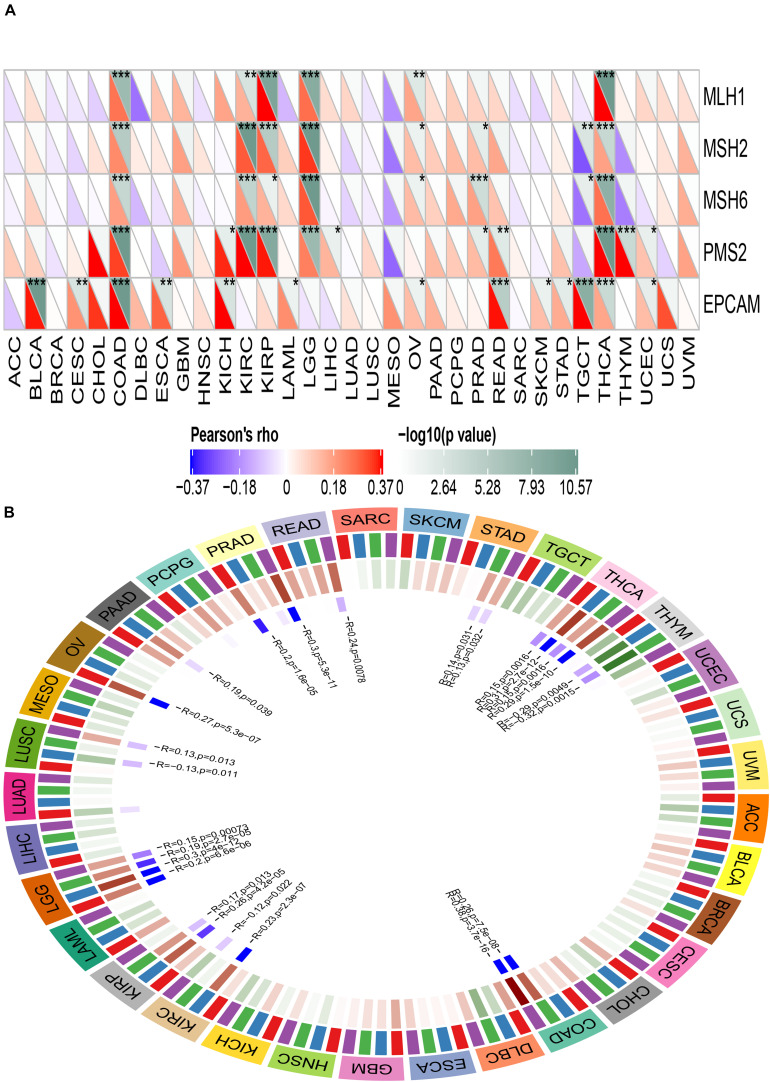
Correlation between ACE2 expression level and Mismatch repair genes **(A)** and DNA methyltransferase **(B)**. *P < 0.05, **P < 0.01; ***P < 0.001.

Mismatch Repair genes are intracellular mismatch repair mechanisms. Mismatch repair deficiency is known to occur in some tumors. TCGA expression profiling was used to evaluate the association between the five key MMRs genes MLH1, MSH2, MSH6, PMS2, EPCAM, and ACE2. The heatmap is shown in Figure 13A. We found that ACE2 was significantly associated with five key MMRs in COAD, KIRC, LGG, TGCT, and THCA.

DNA methyltransferase maintains DNA methylation by transferring methyl groups to the cytosine of CpG dinucleotide islands. DNA hypermethylation in the promoter region of a tumor suppressor gene leads to gene silencing, which in turn leads to dysregulation of multiple signaling pathways associated with human malignancy. The correlation between ACE2 and the expression of four key DNA methyltransferases is shown in Figure 13B. It can be inferred that ACE2 was significantly associated with four key DNMT in THCA, THYM, STAD, PRAD, LUSC, LGG, KIRP, and COAD.

### GSEA Analysis

**FIGURE 14:**
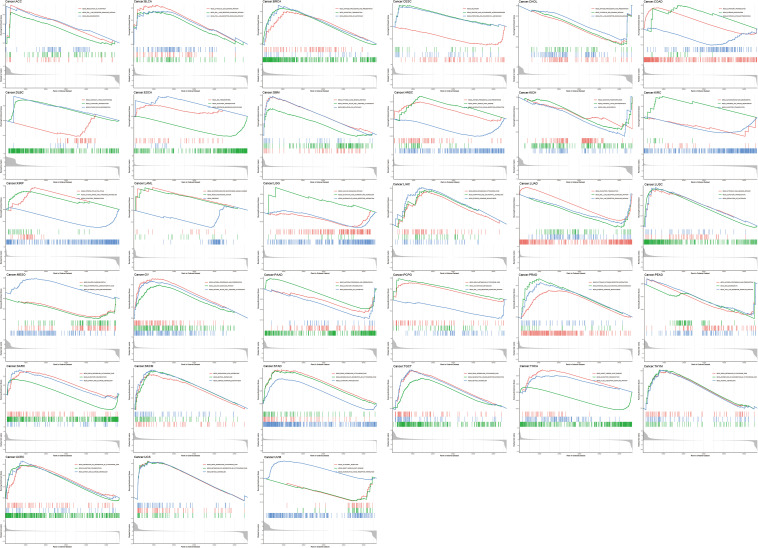
Results of GSEA analysis for the expression of ACE2 across the 31 types of tumors.

To determine the effect of ACE2 on tumors, we divided the samples into high and low groups according to the median of ACE2 expression levels and used GSEA to analyze the enrichment of the KEGG pathways in the high and low expression groups. The first three most significant pathways are shown in Figure 14. The GSEA analysis demonstrated that several cancer-associated pathways and immune-related pathways were hyperactivated in the high ACE2 expression group in most tumors. RIG I LIKE RECEPTOR SIGNALING PATHWAY and the TOLL LIKE RECEPTOR SIGNALING PATHWAY were found in BLCA, ACC, LUAD, and PCPG. REGULATION_OF_AUTOPHAGY was found in ACC, BRCA, GBM, LUSC, and PAAD. NATURAL KILLER CELL MEDIATED CYTOTOXICIT, and T CELL RECEPTOR SIGNALING PATHWAY was found in THCA, GBM, and OV.

## Discussion

The first public reports of SARS-CoV-2 infection, which causes a severe acute respiratory syndrome, were in December 2019. Subsequently, evidence of human-to-human transmission was released in January 2020 (Li et al., 2020). COVID-19 has since caused a global pandemic and the WHO has declared the novel coronavirus disease an international public health emergency. Cancer patients are considered to be highly vulnerable to COVID-19 infection (Liang et al., 2020; Onder et al., 2020). The patient populations with ongoing and previous cancer history represent a unique subset that is more susceptible to SARS-CoV-2 infection, and many cancer patients currently undergoing treatment are greatly concerned about SARS-CoV-2 infection. Recent studies with small cohorts (*n* = 12–28) of SARS-CoV-2 infected cancer patients, have proposed a potentially higher susceptibility and a poorer prognosis for COVID-19 in cancer patients compared to the general population (Liang et al., 2020; Yu et al., 2020; Zhang et al., 2020). A multicenter retrospective cohort study also reported that patients with malignant tumors have a higher risk of developing severe COVID-19 disease (Tian et al., 2020). Moreover, another retrospective study reported that women who received an active treatment for gynecological cancer are at higher risk of severe COVID-19 (Bogani et al., 2020). Yet another study demonstrated that the risk of COVID-19 might outweigh the urgency for cancer treatment (Indini et al., 2020). These findings have indicated that COVID-19 may affect the prognosis of cancer patients. However, there is currently no systematic analysis framework to characterize abnormal ACE2 expression in human cancers.

The Renin-angiotensin system (RAS) is recognized as one of the most important regulators of normal and pathophysiological processes in the brain, kidney, heart, and blood vessels. The activation of the Angiotensin-Converting Enzyme 2/Angiotensin-(1–7)/Mitochondrial Assembly Receptor [ACE2/Ang-(1–7)/MasR] axis is a component of RAS and has recently been identified as an important part of gastric mucosa and cancer. ACE2 appears to play a central role in the formation of Ang-(1–7). ACE2 was linked with fibrosis, inflammation, cancer, angiogenesis (Passos-Silva et al., 2013), and also plays an important role in COVID-19 infection. During viral infection, a key step in the process is the virus entry into the host cells and ACE2-expressing cells are target cells that are susceptible to SARS-CoV-2 infection (Parodi and Liu, 2020). ACE2 appears to play a central role in the ACE2/Ang-(1–7)/MasR axis. A previous meta-analysis has suggested that the ACE2/Ang-(1–7)/MasR axis plays an important role in the development of tumors (de Paula Gonzaga et al., 2020). It is worth noting that ACE2 has both positive and negative roles in tumorigenesis (Xu et al., 2017). On the one hand, ACE2 suppresses breast cancer angiogenesis by inhibiting the VEGF/VEGFR2/ERK pathway (Zhang et al., 2019) and on the other hand, downregulation of the ACE2/Ang-(1–7)/MasR axis enhances breast cancer metastasis by enhancing store-manipulated calcium entry (Yu et al., 2016). Thus, the role of the ACE2/Ang-(1–7)/MasR axis in cancer is complicated.

In this work, a pan-cancer analysis of ACE2 in malignancies in the TCGA dataset was performed. The expression levels of ACE2 and systematic prognostic landscape in different types of cancers were examined using TCGA data. We observed the differential expression of ACE2 between cancer and normal tissues. We found that, compared to normal tissues, ACE2 was highly expressed in colorectal, gastric, kidney, lung, pancreatic cancers, and lymphoma tumors. Some data sets also showed that ACE2 had a lower level of expression in the breast, uterus, brain, skin, ovarian, and prostate cancers. In this work, ACE2 overexpression was found in lung cancer, hepatocellular carcinoma, and gallbladder cancer, which is in line with a previous study (Xu et al., 2017). However, these findings contradict previous reports on ACE2 expression in breast cancer, pancreatic cancer, and oral squamous cell carcinoma (de Carvalho Fraga et al., 2017; Xu et al., 2017). The factors that contribute to these contradictory effects of the ACE2/Ang-(1–7)/MasR axis on cancer require additional investigation.

Although ACE2 was aberrantly expressed in most tumors, its high expression was not associated with OS, DFI, PFI, and DSS, but was associated with KIRC, KIRP, LGG, LIHC, LUSC, OV, UCS, UVM, ACC, COAD, and OV. Interestingly, the high expression level of ACE2 correlated with improved prognosis of KIRC, KIRP, LIHC, LUSC, OV, UCS, UVM, ACC, COAD, and OV, but not LGG. Some reports show that ACE2 may have both positive and negative effects on the development of cancer. It has been demonstrated that overexpressed ACE2 may inhibit cell growth and vascular endothelial growth factor production in lung cancer (Cheng et al., 2016), breast cancer (Yu et al., 2016), colon cancer (Bernardi et al., 2012), and pancreatic cancer (Zhou et al., 2011). On the other hand, overexpressed ACE2 may promote the migration and invasion of human renal carcinoma cells (Zheng et al., 2015; Errarte et al., 2017). The role of ACE2 in cancer development is, therefore, complicated.

It is well-known that the interaction of SARS-CoV-2 with RAS through ACE2 is a key factor in infection. Once SARS-CoV-2 enters the target cell, the host response will be the main determinant of the severity of the subsequent pathogenesis (Gosain et al., 2020; Vaduganathan et al., 2020). Innate lymphocytes including the invariant T (MAIT) cells and γδ Tcells respond quickly to the pathogen invasion and trigger a cytokine response that is essential for the killing of the microorganisms (Hotchkiss and Opal, 2020). After the initial innate response, a specific adaptive immune response is required to eliminate SARS-CoV-2 (Extermann, 2000). Lymphopenia, an independent indicator of poor prognosis in COVID-19 patients, is common and compromises the required immune response (Tan et al., 2020). Long-lasting cytokine release, mediated by more white blood cells than T lymphocytes, may cause a “cytokine storm” and cause severe lung damage (Shi et al., 2020). Hence, another important aspect of this study is that ACE2 expression is correlated with diverse immune infiltration levels in cancer, especially in gastric and colon cancers. Our analyses further demonstrate that ACE2 expression has a significant correlation with the infiltrating levels of dendritic cells, CD4+ T cells, CD8+ T cells, mast cells, B cells, and NK cells in most cancers. ACE2 expression also has a significantly positive correlation with the infiltrating levels of dendritic cells, macrophages M0, mast cells resting, and neutrophils in multiple cancers. ACE2 expression was linked with immune neoantigen, TMB, and microsatellite instability, especially in BRCA and SKCM. Significant correlations were found between ACE2 expression and mismatch repair genes and also with DNA methyltransferases. Thus, our study provides significant insight into the potential role of ACE2 in tumor immunology.

## Conclusion

We found that increased ACE2 expression correlates with immune infiltration levels in most tumors and that higher ACE2 expression was related to immune neoantigen, TMB, and microsatellite instability. These findings indicate that although ACE2 is not associated with prognosis, in most cancers, it is significantly associated with immune penetration levels in a variety of tumors (especially patients with lung and breast cancer) including CD8+ T cells, CD4+ T cells, macrophages, neutrophils, and DC. Our findings confirm that ACE2 affects the immune infiltration of cancer patients with COVID-19. Since this study is only a database analysis, it is necessary to verify the results in a larger clinical cohort.

## Data Availability Statement

The datasets generated for this study can be found in the online repositories. The names of the repository/repositories and accession number(s) can be found in the article/ Supplementary Material.

## Author Contributions

JS, JH, FL, XC, JZ, and XD wrote the main manuscript text. JS and WD prepared Figures 1–14. SQ and JS contributed to data analysis. All authors reviewed the manuscript.

## Conflict of Interest

The authors declare that the research was conducted in the absence of any commercial or financial relationships that could be construed as a potential conflict of interest.

## Footnotes

1 https://portal.gdc.cancer.gov/

2 https://commonfund.nih.gov/GTEx/

3 https://portals.broadinstitute.org/ccle/

4 https://xena.ucsc.edu/

## Supplementary Material

Supplementary Figure 1 Relationship between ACE2 expression level and TNM Stage across most of the tumors.

## Abbreviations

COVID-19: Coronary Virus Disease 2019
ACE2: Angiotensin-converting enzyme 2
SARS-CoV-2: severe acute respiratory syndrome coronavirus 2
ACC: Adrenocortical carcinoma
BLCA: Bladder Urothelial Carcinoma
BRCA: Breast invasive carcinoma
CESC: Cervical squamous cell carcinoma and endocervical adenocarcinoma
CHOL: Cholangiocarcinoma
COAD: Colon adenocarcinoma
DLBC: Lymphoid Neoplasm Diffuse Large B-cell Lymphoma
ESCA: Esophageal carcinoma
GBM: Glioblastoma multiforme
HNSC: Head and Neck squamous cell carcinoma
KICH: Kidney Chromophobe
KIRC: Kidney renal clear cell carcinoma
KIRP: Kidney renal papillary cell carcinoma
LAML: Acute Myeloid Leukemia
LGG: Brain Lower Grade Glioma
LIHC: Liver hepatocellular carcinoma
LUAD: Lung adenocarcinoma
LUSC: Lung squamous cell carcinoma
MESO: Mesothelioma
OV: Ovarian serous cystadenocarcinoma
PAAD: Pancreatic adenocarcinoma
PCPG: Pheochromocytoma and Paraganglioma
PRAD: Prostate adenocarcinoma
READ: Rectum adenocarcinoma
SARC: Sarcoma
SKCM: Skin Cutaneous Melanoma
STAD: Stomach adenocarcinoma
TGCT: Testicular Germ Cell Tumors
THCA: Thyroid carcinoma
THYM: Thymoma
UCEC: Uterine Corpus Endometrial Carcinoma
UCS: Uterine Carcinosarcoma
UVM: Uveal Melanoma.
